# Variations in *AXIN2* predict risk and prognosis of colorectal cancer

**DOI:** 10.1038/s41405-019-0022-z

**Published:** 2019-10-16

**Authors:** L. Otero, E. Lacunza, V. Vasquez, V. Arbelaez, F. Cardier, F. González

**Affiliations:** 1 Dentistry and Sciences Faculties, Center of Dental Research, Pontificia Universidad Javeriana, Carrera 7 No. 40-62, Bogotá, Colombia; 2 Medicine Faculty, Centro de Investigaciones Inmunológicas Básicas y Aplicadas (CINIBA), Universidad Nacional de La Plata, Calle 60 y 120, CP:1900 La Plata, Argentina; 3 Dentistry Faculty, Pontificia Universidad Javeriana, Carrera 7 No. 40-62, Bogotá, Colombia; 4 Gastroenterology, Centro Médico Almirante Colón, Carrera 16. No. 84A-09, Bogotá, Colombia; 5 Dentistry Faculty, Universidad de Cartagena, Cra. 6 #36-100, Cartagena, Bolívar Colombia

**Keywords:** Cancer, Cancer

## Abstract

**Objective:**

Colorectal cancer (CRC) and hypodontia are frequent and different diseases with common genes are involved in their etiology. The objective of this study was to identify the association between AXIN2 rs2240308 with hypodontia and CRC.

**Patients and methods:**

This study consisted of 50 individuals with hypodontia, 50 individuals with CRC, and 155 healthy individuals from Colombia. SNP genotyping assays of rs2240308 were performed and family history of cancer in individuals with hypodontia was documented*.* In silico analysis was implemented to define the genomic profile of the AXIN2 gene associated with CRC. Multivariate analysis, chi square, odd ratio tests, and R software were used for statistical analysis.

**Results:**

AXIN2 rs2240308 showed association with CRC (OR = 5.4 CI: 2.7–10.4; *p* < 0.001) and with other familial cancer in individuals with hypodontia (*p* < 0.005 OR = 1.75, 95% CI: 1.22–6.91). In silico analysis showed that variations in AXIN2 found in CRC patients, were more frequently in earlier stages of tumor and patients who carry variations in the AXIN2 gene have a worse prognosis (*p* < 0.05). The association between AXIN2 rs2240308 with hypodontia was not significant.

**Conclusions:**

These results suggest that AXIN2 rs2240308 polymorphism is associated with CRC and AXIN2 could be a risk marker for predisposition and prognosis of CRC.

## Introduction

Dental agenesis is one of the most common congenital anomalies in human dentition. Hypodontia occurs when there are one to five missing teeth and its prevalence reaches 2.6–11.3% depending the ethnic group.^1^ Although the etiology of dental agenesis involved genetic and environmental factors, the genes more frequently associated with hypodontia in different populations are *AXIN2, MSX1, PAX9, EDA*, and *WNT10*.^2,3^

From the study of Lamni et al.,^4^ many investigations relating dental agenesis with predisposition to cancer, primarily Colorectal cancer (CRC), have arisen.^5–7^ This association is supported by the molecular events that keep homeostasis of morphogenesis and tissue regeneration. CRC is the fourth most common cause of cancer-related deaths in the world ^8^ and it has been reported that colorectal carcinogenesis is associated with alterations in Wnt signaling. Some CRC, like adenomatous polyposis and hereditary nonpolyposis CRC syndrome (Lynch syndrome), involve mutations in germline or in repair genes.^9^ Developmental homeostasis involves the wingless/integration (*WNT*) signaling pathway controlling cell proliferation, differentiation, and cell death.^10^ When cells receive the *WNT* signal, β-catenin is stabilized and joins the DNA-bound T-cell factor family of transcription proteins for regulating the expression of target genes. In the absence of *WNT*, β-catenin protein is degraded by the proteasome via action of a multiprotein complex. This complex is composed of the tumor suppressor adenomatous polyposis coli gene product (APC) and *AXIN1* (axis inhibition protein 1) or its homologous protein, *AXIN2*. They formed a structure with β-catenin, glycogen synthase kinase 3β (GSK3β), and disheveled (DLV) protein.^11^ Hence, molecules such as *WNT (WNT4, WNT6, and WNT10)* and *AXIN2* play an important role during the embryonic development that involves dental formation.^12^

The association between *AXIN2* and CRC involves defects in the canonical *WNT* signaling pathway, which regulates and coordinates the *AXIN* complex for the degradation of β-catenin under normal conditions. In addition, A*XIN*2 expression can be elevated in CRC as a result of APC mutations. However, and in an independent way, alterations in *AXIN2* (loss-of-function, dosage dependent, or even gain-of-function mutations) can contribute to development of gastric cancer.^13^

The genetic connection between alterations in embryonic development of dental organs and predisposition to cancer is understandable; particularly the finding that *AXIN2* mutations could lead to an inefficient block of the *WNT* signaling pathway. Somatic mutations in the AXIN-complex proteins associated with degradation of β-catenin or mutations in β-catenin have been found in different tissues with carcinoma, including skin, gastrointestinal, hepatocellular, and ovarian epithelial cancer.^4,14^ In addition, AXIN2 has also been independently associated with tooth agenesis and non-syndromic cleft lip palate (NSCLP).^15^

*AXIN2* SNP rs2240308 has been mapped at human chromosome 17q23-q24. This polymorphism (rs2240308, c.148 G > A) results in an amino acid change from proline to a serine. Although, rs2240308 has been associated with hypodontia and cancer in different populations,^16,17^ in Iranian subjects, this polymorphism was related with decreased risk for CRC.^18^ The inconsistent results reported in literature are explained by racial differences.^17^ In Latino populations, genetic studies relating hypodontia and CRC are scarce, although *AXIN2* rs2240308 was recently associated with CRC in Mexican population.^19^ Therefore, identifying and analyzing genetic mutations in CRC and hypodontia can provide relevant information about the biological behavior of both diseases. The aim of this study was to identify the association between *AXIN2* rs2240308 with hypodontia and CRC.

## Materials and methods

### Population sample

The population consisted of individuals who assisted to Dentistry faculties at *Pontificia Universidad Javeriana* and *Universidad de Cartagena* and adults who underwent surgery for CRC at gastroenterology private clinic in Bogotá, and *Hospital Universitario del Caribe* in Cartagena, Colombia. Subjects were selected randomly from these institutions and population sample was divided into three groups according to their pathology. A group of 50 subjects with hypodontia, a group of 50 subjects with CRC, and a control group of 155 healthy individuals from both cities in Colombia. This observational analytical cross-sectional study was approved by the Ethical Committees of Dentistry faculty of Pontificia Universidad Javeriana (CIEFOUJ 201108-7539). Informed consent was obtained from all subjects participating.

Patients with hypodontia and healthy individuals were examined at the same clinic by two dentists and patients with CRC were operated by the single professional. Hypodontia-affected individuals were in the age range of 18–28 years. Hypodontia diagnosis was confirmed through complete intraoral examination, panoramic radiographies, and clinical records. Subjects with an uncertain hypodontia diagnosis, syndromes associated with hypodontia, trauma history, or agenesis of third molars were excluded from the study. Subjects with CRC were in the age range of 32–64 years. CRC in all patients was histopathologically confirmed. There was no restriction on sex, age, or histopathological classification and states for selection of CRC patients. The control group included age, sex, and ethnic background matched selected from healthy individuals in the same area during the same time period as the case study. The ethnic background was determined by skin pigmentation and origin of the participants. Subjects with syndromes, hypodontia, trauma, or any type of cancer were excluded.

### Genotyping

Self-reported family history of cancer and hypodontia was collected through questionnaire in all participants (control hypodontia and CRC groups). DNA was obtained from saliva samples through Oragene^®^ DNA kit (DNA Genonek Inc, Canada). *AXIN2* rs2240308 PCR products were obtained from the samples of all the individuals enrolled in the study and sent for genotyping to the Molecular Cloning Laboratories (MCLAB, San Francisco, CA).

### In silico analysis

With the aim of defining the genomic profile of *AXIN2* in CRC, we performed an in silico analysis on data obtained from the web resource cBioPortal. Three comprehensive studies and one TCGA Provisional study were considered: DFCI, Cell Reports 2016 (*n* = 619), Genentech, Nature 2012 (*n* = 276), TCGA, Provisional (Raw data at the NCI, *n* = 633), and MSK, Cancer Cell 2018 (*n* = 1134).^20–22^ The tools provided by cBioportal and R packages from Bioconductor were employed for data integrative analysis and visualization.

For each study, we obtained a network of the most frequently altered neighboring genes of *AXIN2*. The number of genes was filtered according to the percentage of alteration of the neighbor genes, with a set threshold >2.6%. This threshold was established in 2.6 because it is the minimum threshold for some altered gene associated with AXIN2 to appear, at least in the study of Cell Reports 2016. The same procedure was applied to each study, we set the lower threshold in order to obtain altered genes related with AXIN2. To get the overlapping genes between the networks, Euler Diagram was used. APC was the most redundant gene. For gene network association and functional enrichment analysis, we employed the web resources GeneMania (https://genemania.org/) and Enrichr (http://amp.pharm.mssm.edu/Enrichr/), respectively.

### Statistical analysis

The Chi-square, Fisher’s Exact and Odds Ratio (ORs) tests were calculated to assess associations between variations in *AXIN2* rs2240308 with hypodontia and CRC. Multivariate analysis was employed to identify relationship between familial cancer history in patients with hypodontia and *AXIN2* variations. *P* < 0.05 was considered statistically significant. These statistical analyses were performed with and SPSS version 22.0 (software license Pontificia Universidad Javeriana) and in silico analysis was performed with R software (https://www.r-project.org/).

## Results

### Genotyping

The demographic data for the study participants are described in Table 1. Familial cancer history was higher in patients with hypodontia compared with the control group showing a statistical significant difference for *AXIN2* rs2240308 (*p* < 0.005 OR = 1.75, 95% CI: 1.22–6.91). However, Gastric cancer was the most frequent cancer in relatives of patients with hypodontia. In contrast, only 10% of patients with CRC reported family history of hypodontia (Table 2).

**Table 1:** Phenotype for the study participants

Phenotype	CRC group (%) *n* = 50	Hypodontia group (%) *n* = 50	Control group (%) *n* = 155
Age (years)	32–64	18–28	18–64
Sex			
Male	37	23	77
Female	13	27	78
Ethnic background colombia (Latin America)	13 African, 29 American, 8 European	9 African, 29 American, 12 European	19 African, 109 American, 27 European
Origin (place of birth and residence)	25 Cartagena (Caribbean) 25 Bogotá (Central)	25 Cartagena (Caribbean) 25 Bogotá (Central)	65 Cartagena (Caribbean) 90 Bogotá (Central)

Cartagena is located at Caribbean region and Bogotá is located in central zone of Colombia

**Table 2:** Percentage of family history of cancer or hypodontia in all groups (CRC, hypodontia, and control)

Family history of cancer and hypodontia	CRC group (%) *n* = 50	Hypodontia group (%) *n* = 50	Control group (%) *n* = 155
CRC	14	10	1.93
Gastric cancer	8	18	3.22
None	46	38	73.55
Other types of cancer (breast, liver, ovarian, oral, liver, prostatic, lung, brain, kidney)	32	34	21.29
Hypodontia	10	18	2.58

The observed genotype distribution for the Axin2 rs2240308 polymorphism in all groups, controls, hypodontia, and CRC was in agreement with the Hardy–Weinberg equilibrium. Statistical significant association was observed for CRC with *AXIN2* rs2240308 (OR = 5.4, 95% CI: 2.7–10.4; *p* < 0.001). The most frequent type and stage of CRC in this sample was adenocarcinoma (68%) and Dukes'B colon cancer (63.4%).

Regarding to hypodontia, there was no significant association with *AXIN2* rs2240308 (*p* < 0.31). The most frequent tooth agenesis was observed in upper lateral incisors (40%) followed by mandibular second premolars (24%). Other teeth with agenesis were lower lateral incisors, maxillary first premolars, and lower central incisors (36%).

### In silico analysis

**Fig. 1:**
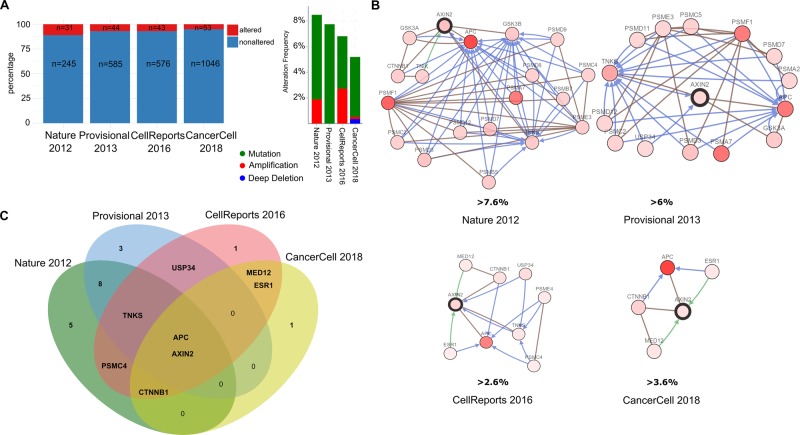
Genomic profile of AXIN2 in colorectal cancer. **a** The percentage and number of samples with gene alterations in AXIN2 are shown across the different datasets included in the analysis. **b** Gene network analysis of AXIN2 associated genes that are also altered in CRC in at least 2.6 % of cases. This percentage—at the bottom of each network—represents the minimum alteration frequency of the genes that integrate the network; below this threshold there are no genes altered associated to AXIN2. **c** Euler diagram of common genes between the different lists of genes obtained from the network analysis

**Fig. 2:**
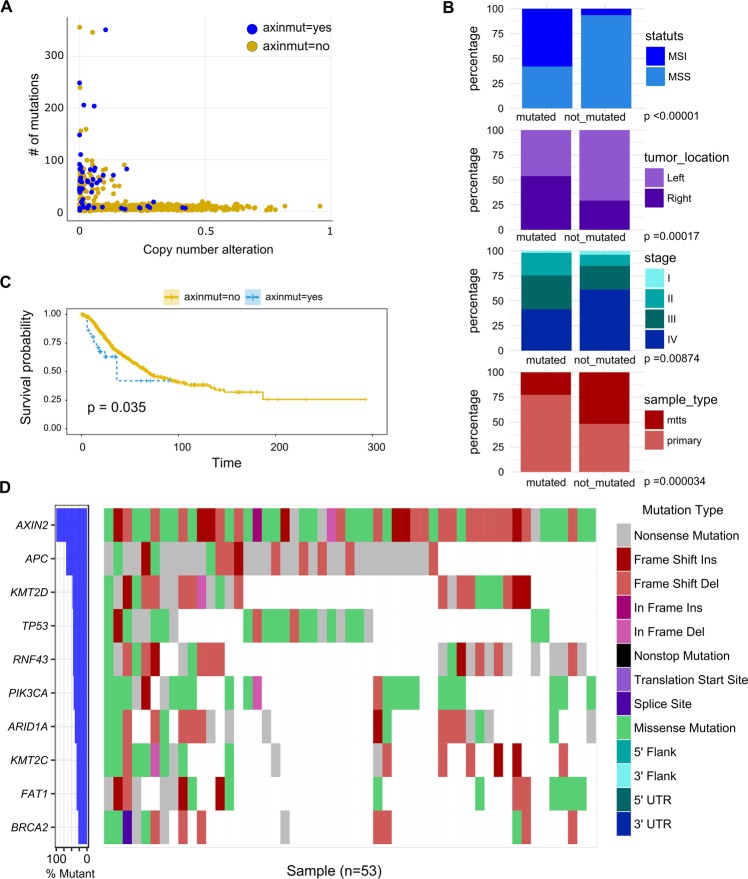
Comprehensive analysis of AXIN2 genomic profile on data from the study of Yaeger et al. **a** AXIN2 mutated samples in the study of Cancer Cell 2018, a total of 53 out of the 1099 patients harbored at least one mutation in AXIN2 gene. **b** Association of clinical variables and AXIN2 mutated samples. **c** Kaplan Meier of the overall survival of patients with AXIN2 mutation. **d** Mutational profile of the most mutated genes in the AXIN2 mutated patients

The genomic profile of *AXIN2* in CRC patients indicates that the frequency of alteration/mutation of the gene is usually not higher than 10% of the patients. It is commonly associated with the alteration of other genes related with the *WNT* pathway, such as *APC* and *CTNBB1*. Genomic profile for *AXIN2* in CRC is shown in Figs. 1 and 2. Also, there was an association with the tumor location, being *AXIN2* gene more frequently mutated in tumor samples derived from the right colon than those derived from the left. Staging and sample type were also evaluated and the group of *AXIN2* mutated showed an association with earlier stages compared with the other group. Interestingly, overall survival analysis indicated that patients who carry variations in the *AXIN2* gene have a worse prognosis (*p* < 0.05).

In summary, by data mining analysis we have defined the genomic profile of *AXIN2* gene in CRC. It is altered in 5–10% of CRC patients, it would be associated with the MSI molecular subtype and right-side tumors. Moreover, *AXIN2* was found more frequently altered in early-stage tumors compared with metastatic CRC. However, patients who harbor mutations in A*XIN2* were found to be associated with a worse prognosis. Along with *AXIN2* those patients also showed mutations in WNT pathway related genes such as *APC, RNF43, PIK3CA*, among others. Mutations in these genes, as well the activation of the *WNT* pathway, have been primarily associated with Instability Microsatellite (MSI) molecular subtype right-side CRC tumors. It is possible that *AXIN2* mutation can be a passenger of these driver genes in MSS tumors, but in turn it could be considered a driver gene in MSI right-side tumors. The association of *AXIN2* mutation with poor prognosis and its appearance in early stages, position it as a prognostic and predictive marker in the defined molecular subtype of right-side colorectal tumors with MSI.^23^

## Discussion

The association between *AXIN2* and CRC has been demonstrated in different populations, but the association between rs2240308 and CRC in Latino American population, it has been previously reported only in Mexican population.^19^ The present study showed a statistically significant association between *AXIN2* rs2240308 and CRC (OR = 5.4 CI: 2.7–10.4; *p* < 0.001). Latin America has a history of large admixture between Africans, Europeans, and Native Americans, for this reason, this region has a high physical and genetic ancestry variation. The Asian ancestry in Colombia and Mexico is <1%.^24^ Significant differences have been reported in the association between rs2240308 and the risk of cancer for type of cancer and ethnic group.^17^ Then, while it has been reported that rs2240308 increased the risk of lung cancer especially in Asian population,^5^ a recent analysis indicated that AXIN2 148 C > T (rs2240308) variant may be associated with decrease lung risk in Asian and Caucasian populations.^25^ Therefore, further studies in Latin American population should be conducted to explain the association between AXIN2 polymorphisms and CRC.

Literature about the association of *AXIN2* rs2240308 polymorphism with cancer show inconsistent results. *AXIN2* rs2240308 polymorphism has been mainly associated with prostate and lung cancer, but its association with ovarian cancer, head and neck cancer, astrocytoma, and CRC did not show similar results. Liu et al.^30^ reported that *AXIN2* is overexpressed in CRC in patients with DNA mismatch repair, but in this study, they did not report the association with rs2240308.^26^ In addition, several studies propose that hypodontia associated with AXIN2 variations could be a risk marker for CRC.^27,28^ In contrast, other studies propose Axin2 rs2240308 as a potential therapeutic target for preventing tumor growth.^29^ The possible explanations for these inconsistencies are related with racial differences observed in these associations^26,30–32^ or, with other gene interactions and gene pathways involved in CRC and in tooth development.^33^

Other possible explanation could be related with the results shown in silico analysis. This analysis demonstrated that the mutation of *AXIN2* observed in CRC is usually not higher than 10% of the patients and it is commonly associated with the alteration of other genes related with the *WNT* pathway. Nonetheless, the most frequently activated signaling in metastatic CRC is the *WNT* pathway. For this reason, we performed additional in silico analysis. This analysis showed that mutations in *AXIN2* found in CRC patients were more frequently in earlier stages of tumor samples derived from the right colon than those derived from the left. Furthermore, patients who carry mutations in the *AXIN2* gene have a worse prognosis (*p* < 0.05). This fact, remark the importance to identify biomarkers for CRC in population, such as *AXIN2* variations in patients with Hypodontia. However, one limitation of our study is the fact that we could not document hypodontia in patients with CRC because we did not have history records of these individuals. Then, we could not establish if the cause of tooth absence was extraction or hypodontia.

Our study showed a statistical significant difference between familial cancer history in individuals with hypodontia and *AXIN2* rs2240308 variations (*p* < 0.005 OR = 1.75, 95% CI: 1.22–6.91). Gastric cancer was the most frequent cancer in this association. The association between AXIN2 and gastric cancer has been reported,^34^ but the association between hypodontia and gastric cancer has not been demonstrated.^15,35^ Predisposition to CRC is reported with greater frequency in patients with severe oligodontia and it is directly related to the loss of *AXIN2* function. In the same context, dental agenesis had been proposed as a risk marker related for ovarian epithelial cancer.^15,36^

According to the results from the present research, upper lateral incisor was the most frequent missing teeth among patients with the *AXIN2* rs2240308, although these associations were not statistically significant. In contrast, *AXIN2* rs2240308 showed association with hypodontia in individuals from Brazil who had at least one missing tooth, although one conclusion of this study was that dental agenesis arises from multiple *AXIN2* gene variants.^37^ Mutations in *AXIN2* have been mainly associated with moderate oligodontia which affects incisors and also individuals with severe oligodontia which is caused by two truncated AXIN2 proteins.^4,38^

In conclusion, the results of the present study showed an association between *AXIN2* rs2240308 and CRC in Colombian population. In silico analysis demonstrated that patients who carry any mutations of AXIN2 have worse prognosis. Future studies should be performed to identify biomarkers in CRC and its association with hypodontia and to identify target molecules for CRC treatment.
